# Utilization of Kenya’s free maternal health services among women living in Kibera slums: a cross-sectional study

**DOI:** 10.11604/pamj.2018.30.86.15151

**Published:** 2018-05-30

**Authors:** Angela Owiti, Julius Oyugi, Dirk Essink

**Affiliations:** 1 Department of Health Sciences, Vrije University, Amsterdam, Netherlands; 2 Institute of Tropical and Infectious Diseases, University of Nairobi, Kenya; 3 Faculty of Earth and Life Sciences, Vrije University, Netherlands

**Keywords:** Maternal health services, delivery services, fee exemption, utilization, slums, Kenya

## Abstract

**Introduction:**

This study was aimed at determining factors affecting utilization of public health facilities by pregnant Kenyan women living in Kibera slums, Nairobi since the implementation of the Free Maternal Service (FMS) Program in 2013.

**Methods:**

This was a cross-sectional study done on 396 women who delivered between 2014 and 2015. Interview questions addressed socio-demographic characteristics, perception of quality of care in public health facilities, awareness of the FMS Program, antenatal care (ANC) and delivery service utilization.

**Results:**

43.9% delivered in a public health facility, 30.3% in a private non-profit health facility (NGO), 22.7% in a private health facility and 3.0% at home. Of the 97% of the women who delivered in a health facility, only 43.9% delivered in a public health facility despite these facilities having free maternal services. Factors that favoured the Free Maternal Service uptake included a positive perception of the public health facility, living within close proximity, learning about the Program from a support group and a short waiting time before being examined by the doctor. On the other hand, safe delivery, quality of service, accessing a health facility on foot, ANC attendance at a private and a non-profit health facility were associated with low uptake of the free maternal services.

**Conclusion:**

The uptake of the Free Maternal Service program can improve if the Kenyan government directs its efforts towards changing women’s perception on quality of care in public health facilities and to improve access to health facilities in slum areas of Nairobi.

## Introduction

Globally, it was estimated that 303,000 maternal deaths occurred in 2015 [1]. Sub-Saharan Africa (SSA) had the highest maternal mortality rate (MMR) at 546 per 100,000 live births compared to the global average of 216 maternal deaths per 100,000 live births [1]. Kenya, according to the World Health Organization (WHO) had one of the highest MMRs at 510 per 100,000 live births. However, the 2014 Kenya Demographic and Health Survey (KDHS) reported a MMR of 362 per 100,000 live births [2].The presence of a skilled birth attendant (SBA) during child birth, readily accessible care in emergency cases and effective systems of communication and referrals, are crucial interventions in improving maternal health [3,4]. Women living in the slums of Nairobi, the capital city of Kenya, were at a disproportionately higher risk of maternal mortality. For instance, the MMR in two of Nairobi’s slums was estimated to be 706 per 100,000 live births [5]. Lack of access to quality maternal care partly contributed to the high maternal risks in these slums [6]. A recent survey showed that 83% of women in these slums delivered at a health facility [7]. However, previous studies documented that slums were mainly served by privately owned and unlicensed informal health facilities with limited skilled personnel and equipment. Most formal health facilities were located outside the slums [8,9]. Other barriers that hindered access to SBAs included poor decision makingat the family level regarding health, limited physical access to formal health facilities, high cost of health services and fear of experiencing negative attitudes from health care workers at formal health facilities [10-12]. The Kenyan government with the goal of increasing access to SBAs, implemented the Free Maternal Service (FMS) Program on June 1, 2013. This new policy exempted women from paying for delivery services at public health facilities [13]. After the introduction of this policy, other researchers studied the barriers to its effective implementation from a healthcare worker’s perspective [14,15]. However, as far as is known, no study has investigated the uptake and access of skilled delivery services in public health facilities by women residing in the slums of Nairobi since the implementation of the FMS Program. Thus the aim of this study was to assess the levels of utilization and factors influencing the uptake of SBAs in public health facilities by expectant women residing in Kibera slums, Nairobi, for delivery since the implementation of the FMS Program.

## Methods

### Study design and setting

This cross-sectional study was conducted in May 2016 in three of the seven administrative geographical sub-locations of Kibera slums in Nairobi, Kenya namely: Gatwekera, Makina and Laini-Saba. Kibera, the largest slum in Kenya has an estimate of approximately 170,078 inhabitants.

### Sample size estimation

Using the estimated proportion of deliveries attended to by SBAs (62%) in Kenya, as reported by the most recent demographic data available at the time of the study [2], the sample size was calculated using the Cochrane formula [16]. The sample size was thus estimated at 363 participants.

### Sampling procedure

A multiple stage sampling technique was used to recruit the 396 study participants. First, the three sub-locations: Gatwekera, Makina and Laini-Saba used to recruit participants, were randomly selected from the seven sub-locations in Kibera. A probability proportional to the population was used to determine the number of women required in each sub-location. Finally, the women to be included in this study were consecutively sampled from their households. Women were eligible if they had lived in Kibera slums for a minimum of one year prior to delivery and had given birth in 2014 or 2015. Where a woman had delivered more than one child between 2014 and 2015, data collection was based on their last delivery. If a household had two women who qualified for the study, the participant was chosenthrough balloting.

### Data collection instrument

The data was collected using a structured questionnaire administered by the researcher and trained research assistants. It was adapted from the household survey carried out by the World Bank and the African Population and Health Research Centre (APHRC) of Nairobi as part of a multi-faceted Maternal Health Study [17,18]. In order to meet the language needs of all the study participants, the questionnaire was prepared in English and Kiswahili (Kenya’s national language) using a forward and backward translation technique. The questionnaire addressed information on socio-demographic characteristics, patterns regarding antenatal care and delivery service utilization, women’s perception of quality of care in public health facilities, and the women’s awareness of the FMS Program. Prior to data collection, pretesting of the questionnaire was done in other unselected sub-locations. The principal investigator and a field supervisor closely followed the data collection process and checked for completeness and consistency of the questionnaires.

### Variables

The dependent variable of interest was the dichotomous variable; utilization of public health facilities for delivery. Alternatives to using public health facilities include: utilization of a private health facility, private non-profit health facility (NGO) or home delivery.

The independent variables for this study were grouped as knowledge of the FMS Program, accessibility and perceived quality of care. Knowledge of the FMS Program was assessed based on: awareness, source of information regarding the program, attending ANC and where one attended ANC. Accessibility was assessed on: travel time to the health facility, the mode of transport used, difficulty of access to the transport mode and the waiting time between first arriving at the health facility and being examined by the nurse/doctor. Perceived quality of care was based on the participant’s opinions of the public health facility and the quality of care given by the doctors and nurses in regards to service delivery at the closest public health facility. The participant’s perceptions were measured using a set of 9 Likert scale questions categorized as: don’t know (0), poor perception (1), neutral (2) and positive perception (3). Thus, 27 (9x3) was the maximum score attainable. The participant’s final score was then computed and one’s perceived quality of care of the mentioned public health facility was categorized as follows: having a low perception (score of 0-9), a neutral perception (score of 10-18) or a positive perception (score of 19-27).

Although not a key independent variable, the potential influence of socio-demographic characteristics on a woman’s place of delivery, was included in the analysis to give a more comprehensive picture. Socio-demographic characteristics were assessed on: age, religion, marital status, number of children, education level, employment and income level.

### Statistical analysis

Statistical Package for Social Sciences (SPSS) versions 21 was used to analyze data. To present the women’s characteristics and their use of delivery care, descriptive statistics was used. The association between the dependent and independent variables was assessed using bivariate and multivariate analysis. The former was performed using chi-square and logistic regression analysis tests. The confidence interval (CI) was set at 95% and results considered significant at a *p* value of < 0.05. For the logistic regression analysis, the odds ratio was also taken into account. To control for potential confounders, independent variables found to be significant at the bivariate analysis were included in some multiple logistic regressions.

### Ethical clearance

Ethical approval for this study was obtained from the University of Nairobi and Kenyatta National Hospital’s Ethics Review Committee. Informed consent was obtained from all participants after full explanation of the study design and purposes. In cases where a participant was below 18 years, consent was obtained from the parents/guardians.

## Results

### Socio-demographic characteristics of women who delivered in 2014 and 2015 in Kibera slums

The enrolled women in the study were mainly in the 20-34 years age bracket, of Christian faith, married and literate. About 30% of these women had one child, 35% had two children and 20.3% had three children. Majority of the respondents were unemployed. Of those employed, 18.7% earned between 0 and 5,000 Kenyan Shillings and 14.9% earned between 5,001 and 10,000 Kenyan Shillings (Table 1).

**Table 1:** The influence of socio-demographic characteristics on the utilization of public health facilities

	Total	Utilization of public health facilities		
Variables	n (%)	Yes = n (%)	No = n (%)	p value
**Location**				0.248
Makina	251(63.4)	106(42.2)	145(57.8)	
LainiSaba	85(21.5)	44(51.8)	41(48.2)	
Gatwikira	60(15.2)	24(40.0)	36(60.0)	
**Age**				0.267
< 20 years	26(6.6)	12(46.2)	14(53.8)	
20 - 34 years	325(82.1)	148(45.5)	177(54.5)	
≥ 35 years	43(10.9)	14(32.6)	29(67.4)	
**Religion**				0.242
Christian	301(77.2)	138(45.8)	163(54.2)	
Islam	87(22.0)	34(39.1)	53(60.9)	
None	2(0.5)	0(0.0)	2(100.0)	
**Marital Status**				0.045
Single	86(21.7)	42(48.8)	44(51.2)	
Married	291(73.5)	118(40.5)	173(59.5)	
Living together	1(0.3)	1(100.0)	0(0.0)	
Divorced	16(4.0)	12(75.0)	4(25.0)	
Widowed	2(0.5)	1(50.0)	1(50.0)	
**Number of children**				0.444
0	3(0.8)	1(33.3)	2(66.7)	
1	121(30.7)	60(49.6)	61(50.4)	
2	138(35.0)	56(40.6)	82(59.4)	
3	80(20.3)	30(37.5)	50(62.5)	
4	30(7.6)	14(46.7)	16(53.3)	
≥ 5	22(5.6)	12(54.5)	10(45.5)	
**Education level**				0.162
Primary	191(48.2)	77(40.3)	114(59.7)	
Secondary	174(43.9)	85(48.9)	89(51.1)	
College	27(6.8)	9(33.3)	18(66.7)	
University	2(0.5)	1(50.0)	1(50.0)	
None	2(0.5)	2(100.0)	0(0.0)	
**Occupation**				0.227
No work	249(62.9)	105(42.2)	144(57.8)	
Farming	2(0.5)	2(100.0)	0(0.0)	
Trading/Selling	46(11.6)	18(39.1)	28(60.9)	
Office work	12(3.0)	7(58.3)	5(41.7)	
Student	2(0.5)	0(0.0)	2(100.0)	
Labourer/Casual worker	85(21.5)	42(49.4)	43(50.6)	
**Income**				0.795
0 - 5,000	74((18.7)	36(48.6)	38(51.4)	
5,001 - 10,000	59(14.9)	27(45.8)	32(54.2)	
10,001 - 20,000	6(1.5)	3(50.0)	3(50.0)	
20,001 - 30,000	3(3)	2(66.7)	1(33.3)	
> 30,000	1(1)	0(0.0)	1(100.0)	
Refusetoanswer		0(0.0)	1(100.0)	

P < 0.05 = significant > 0.05 = not significant; p values derived from chi square tests

### Place of delivery

**Figure 1:**
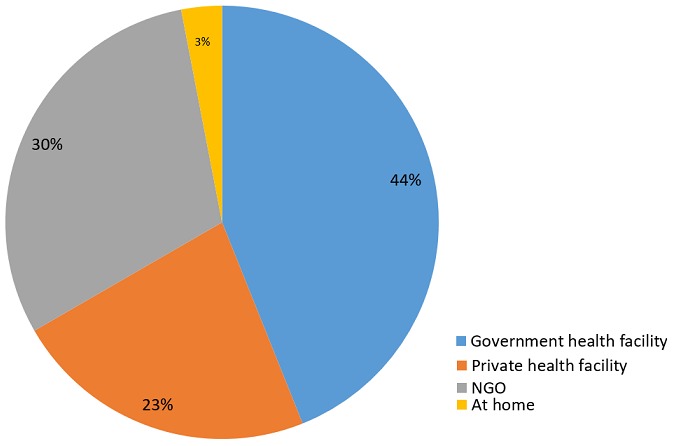
Percentage distribution of place of delivery of women who gave birth in 2014 and 2015 in Kibera

A total of 396 women who had given birth in 2014 and 2015 were enrolled in the study. The results showed that 384 (97%) delivered at a health facility. Of the total respondents, 174 (43.9%) delivered at a public health facility, 120(30.3%) at an NGO health facility, 90 (22.7%) at a private health facility and 12(3.0%) delivered at home (Figure 1). Of the 12 women who delivered at home, 11 had planned a health facility delivery, of which 3 in a public one, 2 in a private one and 6 at an NGO health facility.

### Main reasons for not giving birth in a public health facility

Long distance from the public health facility (42.6%), perceived poor quality of care (23.8%) and negative attitude of the health care workers’ (15.8%) were among the reasons advanced by women for not using public health facilities for delivery. Another 13.9% cited other reasons including fear of: being charged for delivery, child being stolen from facility, and death of baby as experienced during previous delivery.

### Bivariate analysis for the utilization of skilled birth attendants in public health facilities

The results of the bivariate analysis (Table 1), indicated that marital status was significantly linked to the utilization of public health facilities. As shown in Table 2, women who had a complication during their most recent pregnancy (p < 0.0001), also women who had complications during the previous pregnancy (p < 0.0001), also women who were referred during labour (p < 0.0001) had a higher utilization of public health facilities compared to private health facilities (NGO or private for profit health facilities). Women who chose a health facility because they expected good quality of services (p < 0.0001) and because it was near (p < 0.0001), had a higher utilization of private health facilities (NGO or private for profit health facilities).

**Table 2:** Reason for choosing place of delivery

	Utilization of public health facilities		
Variable	Yes = n (%)	No = n (%)	p value
**Nurse/doctor told you**			
Yes	5 (62.5)	3 (37.5)	0.324
No	169 (44.9)	207 (55.1)	
**For safe delivery**			
Yes	18 (16.5)	91 (83.5)	< 0.0001
No	156 (56.7)	119 (43.3)	
**Complications during this most recent pregnancy**			
Yes	30 (96.8)	1 (3.2)	< 0.0001
No	144 (40.8)	209 (59.2)	
**Complications during the previous pregnancy**			
Yes	19 (90.5)	2 (9.5)	< 0.0001
No	155 (42.7)	208 (57.3)	
**Baby was overdue**			
Yes	0 (0.0)	2 (100.0)	0.197
No	174 (45.5)	208 (54.5)	
**Referred during labour**			
Yes	51 (98.1)	1 (1.9)	< 0.0001
No	123 (37.0)	209 (63.0)	
**Husband/relative asked me**			
Yes	3 (75.0)	1 (25.0)	0.231
No	171 (45.0)	209 (55.0)	
**Good quality of service**			
Yes	27 (21.4.)	99 (78.6)	< 0.0001
No	147 (57.0)	111 (43.0)	
**Health facility is near**			
Yes	31 (20.1)	123 (79.9)	< 0.0001
No	143 (62.2)	87 (37.8)	
**Because it is free**			
Yes	57 (47.5)	63 (52.5)	0.562
No	117 (44.3)	147 (55.7)	

p < 0.05 = significant, p > 0.05 = non-significant; p values derived from chi square tests

Table 3 depicted that awareness of the FMS Program was not significantly associated with utilization of public health facility delivery. Women who attended ANC at a public health facility had a higher utilization of public health facilities for delivery compared to women who attended ANC at a private or an NGO health facility (p < 0.0001). The results in Table 4 showed that delivery in a public health facility was more likely if women had a positive perception (OR = 4.9, 95% CI = 2.5-9.7) of public health facilities within their closest proximity.

**Table 3:** Knowledge of the FMS Program and the utilization of public health facilities

	Utilization of public health facilities		
Variable	Yes-n%	No = %	p value
**ANC visits**			0.258
Yes	173 (43.8)	222 (56.2)	
No	1 (100.0)	0 (0.0)	
**Where one attended ANC**			< 0.0001
Traditional birth attendant	0 (0.0)	0 (0.0)	
Public health facility	77 (65.8)	40 (34.2)	
Private health facility	15 (24.6)	46 (75.4)	
NGO health facility	81 (37.3)	136 (62.7)	
**Number of ANC visits**			0.119
1 time	2 (66.7)	1 (33.3)	
2 times	5 (55.6)	4 (44.4)	
3 times	18 (34.6)	34 (65.4)	
4 times	69 (39.2)	107 (60.8)	
> 4 times	77 (50.7)	75 (49.3)	
**Awareness of the FMS Program**			
Yes	163 (44.1)	207 (55.9)	0.862
No	11 (42.3)	15 (57.7)	
**Source of Information**			
**Mass media**			
Yes	126 (43.4)	164 (56.6)	0.655
No	37 (46.3)	43 (53.8)	
**Husband/relatives**			
Yes	15 (62.5)	9 (37.5)	0.060
No	148 (42.8)	198 (57.2)	
**Friends**			
Yes	53 (42.4)	72 (57.6)	0.647
No	110 (44.9)	135 (55.1)	
**Hospital Staff**			
Yes	41 (43.2)	54 (56.8)	0.838
No	122 (44.4)	153 (55.6)	
**Community Health Volunteer**			
Yes	17 (47.2)	19 (52.8)	0.687
No	146 (43.7)	188 (56.3)	
**Religious leaders**			
Yes	0 (0.0)	0 (0.0)	-
No	163 (44.1)	207 (55.9)	
**NGO/CBO**			
Yes	4 (50.0)	4 (50.0)	0.732
No	159 (43.9)	203 (56.1)	
**Support group**			
Yes	176 (72.7)	6 (27.3)	0.005
No	147 (42.2)	201 (57.8)	

P < 0.05 = significant; p values derived from chi square test

**Table 4:** The influence of perceived quality of care on the utilization of public health facilities

Perception category	Utilization of public health facilities		OR [95% CI of OR]	p value
	Yes = n (%)	No = (n %)		
Low	15 (24.6)	46 (75.4)	ref	
Neutral	74 (38.1)	120 (61.9)	1.9 [1.0-3.6]	0.055
Positive	85 (61.6)	53 (38.4)	4.9 [2.5-9.7]	< 0.0001

P < 0.05 = significant; OR = odds ratio; CI = Confidence Interval; ref = reference category. Dependent variable: utilization of public health facilities

The results in Table 5 showed that the time taken to get to the health facility, the waiting time between arrival at the health facility and before examination by the doctor/nurse and mode of transport used to get to the health facility, was significantly associated with the utilization of public health facilities (p < 0.0001). The study results showed that at 47%, walking to a health facility was the most common means of transport used by the women (Data not shown). Only 15.3% of these women who travelled by foot delivered at a public health facility compared to 84.7% of women who delivered in either a private or an NGO health facility (p < 0.0001).

**Table 5:** Factors associated with access to and use of public health facilities

Factors affecting access of public health facilities	Utilization of public health facilities		p value
	Yes = n (%)	No = n (%)	
**Time taken to get to health facility**			< 0.0001
>30 minutes	76 (36.2)	134 (63.8)	
30 minutes - 1hour	78 (52.7)	70 (47.3)	
1hour-2hours	13 (68.4)	6 (31.6)	
<2hours	5 (100.0)	0 (0.0)	
Don’t know	2 (100.0)	0 (0.0)	
**Mode of transport to the health facility?**			< 0.0001
Private car	25 (65.8)	13 (34.2)	
Matatu	46 (83.6)	9 (16.4)	
Taxi	25 (69.4)	11 (30.6)	
Bicycle	3 (100.0)	0 (0.0)	
Motorcycle	12 (41.4)	17 (58.6)	
Ambulance	34 (100.0)	0 (0.0)	
On foot	29 (15.3)	160 (84.7)	
**Waiting time before being examined**			< 0.0001
>30minutes	126 (39.4)	194 (60.0)	
30minutes-1 hour	32 (76.2)	10 (23.8)	
<1 hour	15 (78.9)	4 (21.1)	
Don’t know	1 (100.0)	0 (0.0)	

P < 0.05 = significant; p values derived from chi square test

### Multivariate analysis for the utilization of skilled birth attendants in public health facilities

Table 6 illustrated the independent variables that were predictors of the utilization of SBAs in public health facilities. After adjusting for the following variables: marital status, choosing a health facility because of complication(s) during the most recent pregnancy and the previous pregnancy and time taken to get to the health facility, the multivariate analysis showed that women with a positive perception of the public health facility within closest proximity (OR = 17, 95% CI: 4.5-66.6) were 17.3 times more likely to deliver at a public health facility than those with a low perception. Women who attended ANC at a private health facility (OR = 0.049, 95% CI: 0.012-0.196) and an NGO health facility (OR = 0.081, 95% CI: 0.028-0.235) were less likely to deliver at a public health facility.

**Table 6:** Logistic regression model showing the coefficient and OR of factors associated with utilization of public health facilities

Variables	Coefficient	S.E. for Coefficient	p value	OR	95% C.I. for OR	
					Lower	Upper
**Perception about public health facility delivery**						
0-9 (low perception)	ref					
10-18 (neutral)	1.029	0.694	0.138	2.799	0.718	10.914
19-27 (positive perception)	2.852	0.688	<0.001	17.315	4.499	66.640
**Where attended ANC**						
Public health facility	ref					
Private health facility	-3.013	0.705	< 0.001	0.049	0.012	0.196
NGO	-2.508	0.540	< 0.001	0.081	0.028	0.235
**Reason for choosing place of delivery**						
For safe delivery	-2.272	0.548	< 0.001	0.103	0.035	0.302
Good quality of service	-1.818	0.494	< 0.001	0.162	0.062	0.427
Health facility is near	-2.070	0.472	< 0.001	0.126	0.050	0.318
**Means of transport to the facility**						
Private car	ref					
Matatu	-.211	0.810	0.795	0.810	0.166	3.959
Taxi	-.226	0.820	0.783	0.798	0.160	3.980
Bicycle	21.283	21473.378	0.999	-		
Motorcycle	-0.267	0.892	0.765	o.766	0.133	4.398
Ambulance	19.628	7025.325	0.998	-		
On foot	-2.585	0.693	< 0.001	0.075	0.019	0.293
**Waiting time at the facility**						
Less than 30minutes	ref					
30 minutes to 1 hour	2.117	0.652	<0.001	8.310	2.313	29.850
More than 1 hour	2.935	0.995	0.003	18.825	2.679	132.300
Don’t know	0.476	40802.329	1.000	-		
Source of information						
Support group	2.862	0.952	0.003	17.505	2.708	113.146

P < 0.05 = significant. Dependent variable: Utilization of public health facilities. Ref = Reference category. OR = odd ratio. CI = confidence interval

Those women who reported that their choice of health facility was facilitated by need of a safe delivery (OR = 0.103, 95% CI: 0.035-0.302) because of good quality of service (OR=0.162, 95% CI: 0.062-0.427) and because the facility is near (OR=0.126, 95% CI: 0.050-0.318), were less likely to deliver at a public health facility. Regarding mode of transport, women who reported travelling by foot were less likely to deliver at a public health facility (OR= 0.075, 95% CI: 0.019-0.293). Women who reported waiting more than 30 minutes (OR = 8.310, 95% CI: 2.313-29.850) and waiting between 30minutes to 1 hour (OR = 18.825, 95% CI: 2.679-132.300) before being examined by a doctor/nurse, were more likely to deliver at a public health facility. Women who reported that they received information of the FMS Program from a support group were 17.5times more likely to deliver at a public health facility (OR = 17.5, 95% CI: 2.708-113.146).

## Discussion

The study assessed utilization of public health facilities since the implementation of the FMS Program. Considering the most recent delivery a mother had, the study showed that 97% of women delivered in a health facility (public, NGO or private health facility). This estimate was not only higher than the 88.5% observed in a 2012 Nairobi Cross-sectional Slums Survey [7], but also the national average (62%) [2]. This could imply that the fee exemption policy offered by the Kenyan government encourages women to deliver in health facilities, whilst reducing cost barriers as observed in other developing countries, Ghana and Laos [19,20]. However, only 43.9% of the women chose to deliver in a public health facility. The results of our study showed that utilization of public health facilities was significantly higher among women who were referred or had had complications during their most recent or previous pregnancies. The authors further noted that NGO health facilities also offered free delivery services in Kibera slums. This could have deterred some women from seeking delivery care in public health facilities. In contrast to a previous study on user fees which found that women were more likely to seek professional delivery care when they are aware of the free delivery services offered [21], this study suggests otherwise. This is because there was no statistically significant association with the use of SBAs in public health facilities and awareness of the FMS Program. With 90% of women conscious of the FMS Program, it could indicate that other factors account for majority of the women choosing not to deliver in public health facilities.

Perceived low quality of care is a major barrier to the utilization of maternal health services and can lead to a first delay in deciding to seek care [22-24]. This analysis revealed that women with a positive perception of public health facilities had a significantly increased likelihood of delivering at the same in comparison to women with a negative perception. Additionally, women who chose a health facility because they expected good quality of services and a safe delivery, were more likely to deliver at a private or NGO health facility. Previous research and reports have addressed the issue of poor quality of care and shown that public health workers are often disrespectful, unfriendly and neglectful [25-27]. This issue of poor quality of care may be linked to a generalized problem of the healthcare delivery system [25].

Our results on factors affecting access to public health facilities, showed that majority of the women accessed the health facility on foot which was found to significantly decrease the likelihood of delivering at a public health facility. Previous qualitative studies in similar contexts have found that poor road networks within Nairobi slums made transportation facilities inaccessible [8,10]. The inability to access appropriate means of transportation during labour may have deterred a significant proportion of women from accessing public health facilities for delivery. Additionally, the limited availability of public health facilities in the slums [28] likely contributes to the way in which the private sector is meeting the women’s need for maternal health care. Moreover, women who mentioned choosing a health facility because it was in close proximity, showed an association with delivering at NGO or private health facilities. This study however, did not measure the actual physical distance to assess how it affects women’s access to delivery services in public health facilities. Moreover, longer waiting times was significantly associated with public health facility delivery utilization. Perhaps women who are aware of the longer waiting times in public health facilities are less inclined to seek professional delivery care in these facilities.

Further research conducted on the policy is recommended to evaluate the women’s’ satisfaction level with the FMS program. Future research should identify whether women are satisfied with the maternal service provisions at public health facilities and whether they would return for future delivery or recommend someone to deliver there.

### Strengths and limitations of the study

As far as is known, this was the first analysis that determined the levels of utilization of SBAs in public health facilities and assessed the factors that influenced its utilization since the implementation of the FMS Program. One of the limitations in this cross-sectional study, was the inability to provide a cause-effect relationship. Recall bias was a potential limitation since the data collected was self-reported by the women. However, it is hoped that this may not have been an issue because recall bias is less likely with issues regarding pregnancy, which are viewed as less sensitive [29]. In addition, there was no validation of the information provided by the women. Data was collected by community health volunteers who are known within the community and, this could have led the women to provide sociable desirable answers leading to bias. Some eligible candidates such as women who work, may have been missed during the sampling process but, attempt was made to regulate this by collecting data during different times of the day. Moreover, women who delivered at home or lost their child during delivery, could have refused to participate in the study.

## Conclusion

This study showed that the proportion of women in the study area utilizing health facilities for deliveries was very high. However, the free maternal service offered by the Kenyan government is not sufficient alone to attract women living in Kibera slums to utilize public health facilities. Mostly because of poor perceived quality of services and constraints of access factors. Hence, the private health facilities will continue to play a major role in providing maternal delivery services to expectant women. There is a need for the Kenyan government to partner with the non-public health sector with the goal of increasing access to SBAs. Previous studies have shown that some of the private health facilities operating in the slums do not always meet the basic standards of obstetric care. Therefore, the partnership should strive to ensure the delivery of quality maternal health services. Public health facilities could attract more clients by improving the effectiveness in which they work and overall customer care relations.

### What is known about this topic

- Kenya has a high maternal mortality rate;
- Provision of skilled delivery plays a crucial role in reducing maternal mortality;
- Cost is considered a hindrance to the utilization of skilled birth attendants.

### What this study adds

- Women living in Kibera slums, Nairobi, are aware of the importance of delivering with a skilled birth attendant;
- Offering free maternal services is not enough motivation for some women to use the services;
- Factors such as perceived quality of care and accessibility are also key indicators.

## References

[1] World Health Organization, UNICEF Trends in maternal mortality: 1990-2015: estimates from WHO, UNICEF, UNFPA, World Bank Group and the United Nations Population Division: executive summary.

[2] Kenya National Bureau of Statistics (KNBS) and ICF International (2015). Kenya Demographic and Health Survey 2014: key indicators.

[3] Adegoke A, van den Broek N (2009). Skilled Birth Attendance- Lessons Learnt. BJOG: An International Journal of Obstetrics & Gynaecology.

[4] Koblinsky M, Matthews Z, Hussein J (2006). Going to scale with professional skilled care. The Lancet.

[5] Ziraba K, Madise N, Mills S (2009). Maternal mortality in the informal settlements of Nairobi city: what do we know?. Reprod Health.

[6] Ziraba K, Mills S, Madise N (2009). The state of emergency obstetric care services in Nairobi informal settlements and environs: results from a maternity health facility survey. BMC Health Services Research.

[7] African Population and Health Research Center (APHRC). 2014 (2012). Population and Health Dynamics in Nairobi’s Informal Settlements: Report of the Nairobi Cross-sectional Slums Survey (NCSS.

[8] Fotso J, Ezeh A, Oronje R (2008). Provision and use of maternal health services among urban poor women in Kenya: what do we know and what can we do?. J Urban Health.

[9] Fotso J, Ezeh A, Madise N (2009). What does access to maternal care mean among the urban poor? Factors associated with use of appropriate maternal health services in the slum settlements of Nairobi, Kenya. Maternal and Child Health Journal.

[10] Essendi H, Mills S, Fotso J (2011). Barriers to formal emergency obstetric care services’ utilization. J Urban Health.

[11] Izugbara C, Kabiru C, Zulu E (2009). Urban poor kenyan women and hospital-based delivery. Public Health Reports.

[12] Izugbara C, Ngilangwa P (2010). Women, poverty and adverse maternal outcomes in Nairobi, Kenya. BMC Womens Health.

[13] Ministry of Health, Kenya (2015). Status of Implementation of Free Maternity Services(FMS) program in the Devolved Health System in Kenya. A Comprehensive Assessment Report.

[14] Lang’at E, Mwanri L (2015). Healthcare service providers’ and facility administrators’ perspectives of the free maternal healthcare services policy in Malindi District, Kenya: a qualitative study. Reprod Health.

[15] Wamalwa E (2015). Implementation challenges of free maternity services policy in kenya: the health workers’ perspective. The Pan African Medical Journal.

[16] Cochran WG (1977). Sampling Techniques- 3rd edition.

[17] Bazant E (2008). Women's place of delivery and experience of quality in delivery care: a quantitative and qualitative study in Nairobi's informal settlements.

[18] Mills S, Bos E, Lulu E (2007). Obstetric care in poor settings in Ghana, India and Kenya.

[19] Boudreaux C, Chanthala P, Lindelow M (2014). Assessing the elimination of user fees for delivery services in Laos. PLoS ONE.

[20] Dzakpasu S, Soremekun S, Manu A (2012). Impact of free delivery care on health facility delivery and insurance coverage in Ghana’s Brong Ahafo Region. PLoS ONE.

[21] Mills S, Williams J, Adjuik M (2008). Use of health professionals for delivery following the availability of free obstetric care in Northern Ghana. Maternal and Child Health Journal.

[22] Ganle JK, Parker M, Fitzpatrick R, Otupiri E (2014). A qualitative study of health system barriers to accessibility and utilization of maternal and newborn healthcare services in Ghana after user-fee abolition. BMC Pregnancy Childbirth.

[23] Silan V, Kant S, Archana S (2014). Determinants of underutilisation of free delivery services in an aarea with high institutional delivery rate: a qualitative study. North American Journal of Medical Sciences.

[24] Thaddeus S, Maine D (1994). Too far to walk: maternal mortality in context. Soc Sci Med.

[25] Bourbonnais N (2013). Implementing free maternal health care in Kenya: challenges, strategies, and recommendations.

[26] Fotso J, Mukiira C (2012). Perceived quality of and access to care among poor urban women in Kenya and their utilization of delivery care: Harnessing the potential of private clinics?. Health Policy and Planning.

[27] Ojwang BO, Ogutu EA, Matu PM (2010). Nurses' impoliteness as an impediment to patients' rights in selected Kenyan hospitals. Health Hum Rights.

[28] Rossier C, Muindi K, Soura A (2014). Maternal health care utilization in Nairobi and Ouagadougou: evidence from HDSS. Glob Health Action.

[29] Babalola S, Fatusi A (2009). Determinants of use of maternal health services in Nigeria - Looking beyond individual and household factors. BMC Pregnancy and Childbirth.

